# SarcoNet: A Pilot Study on Integrating Clinical and Kinematic Features for Sarcopenia Classification

**DOI:** 10.3390/diagnostics15192513

**Published:** 2025-10-03

**Authors:** Muthamil Balakrishnan, Janardanan Kumar, Jaison Jacob Mathunny, Varshini Karthik, Ashok Kumar Devaraj

**Affiliations:** 1 Department of Biomedical Engineering, SRM Institute of Science and Technology—Kattankulathur, Chengalpattu 603203, India; pearl.tamil11@gmail.com (M.B.); jaisjac@gmail.com (J.J.M.); varshink@srmist.edu.in (V.K.); 2 Department of General Medicine, SRM Medical College Hospital and Research Centre, SRM Institute of Science and Technology—Kattankulathur, Chengalpattu 603203, India; kumarj1@srmist.edu.in

**Keywords:** motion analysis, artificial Intelligence, joint angles, machine learning

## Abstract

**Background and Objectives:** Sarcopenia is a progressive loss of skeletal muscle mass and function in elderly adults, posing a significant risk of frailty, falls, and morbidity. The current study designs and evaluates SarcoNet, a novel artificial neural network (ANN)-based classification framework developed in order to classify Sarcopenic from non-Sarcopenic subjects using a comprehensive real-time dataset. **Methods:** This pilot study involved 30 subjects, who were divided into Sarcopenic and non-Sarcopenic groups based on physician assessment. The collected dataset consists of thirty-one clinical parameters like skeletal muscle mass, which is collected using various equipment such as Body Composition Analyser, along with ten kinetic features which are derived from video-based gait analysis of joint angles obtained during walking on three terrain types such as slope, steps, and parallel path. The performance of the designed ANN-based SarcoNet was benchmarked against the traditional machine learning classifiers utilised including Support Vector Machine (SVM), k-Nearest Neighbours (k-NN), and Random Forest (RF), as well as hard and soft voting ensemble classifiers. **Results:** SarcoNet achieved the highest overall classification accuracy of about 94%, with a specificity and precision of about 100%, an F1-score of about 92.4%, and an AUC of 0.94, outperforming all other models. The incorporation of lower-limb joint kinetics such as knee flexion, extension, ankle plantarflexion and dorsiflexion significantly enhanced predictive capability of the model and thus reflecting the functional deterioration characteristic of muscles in Sarcopenia. **Conclusions:** SarcoNet provides a promising AI-driven solution in Sarcopenia diagnosis, especially in low-resource healthcare settings. Future work will focus on improving the dataset, validating the model across diverse populations, and incorporating explainable AI to improve clinical adoption.

## 1. Introduction

Sarcopenia is an age-related progressive skeletal muscle disease characterised by accelerated loss of skeletal muscle mass and function, leading to falls, fractures, physical disabilities, and death [1]. Early identification of the risk of Sarcopenia in geriatric patients is essential for providing necessary intervention strategies and improving patient health. Although it is believed that ageing is the primary contributory factor, other factors including a reduction in the nerve cells that transmit signals from the brain to the muscles, a drop in hormone levels, a decline in the body’s capacity to convert protein into energy, and a failure to consume enough protein and calories each day to maintain muscle mass are some of the factors that cause Sarcopenia [2]. The ageing of the world’s population is expected to lead to an increase in the prevalence of Sarcopenia, which will lead to an increase in the cost of medical care and a decrease in the quality of life for those who are affected by it.

According to the European Working Group on Sarcopenia in Older People (EWGSOP), Sarcopenia is estimated to be around 1.6% in men aged 40–79 years and 3.66% in the geriatric population greater than 85 years [3,4]. It has been estimated that 39.2% of Indians suffer from primary Sarcopenia [5]. Research indicates that 53.95% of the elderly population in South India is afflicted by sarcopenia [6]. Assessing muscle mass is crucial for diagnosing Sarcopenia. The diagnosis of Sarcopenia was based on skeletal muscle mass in the limbs divided by the square of height (kg/m^2^), falling below 2.0 standard deviations of the mean for the younger population [7]. A decrease in muscle mass defines Sarcopenia, and hence the assessments of muscle mass in individuals with Sarcopenia encompass magnetic resonance imaging (MRI) [8], computed tomography enterography (CTE) [9], dual-energy X-ray absorptiometry (DEXA), and bioelectrical impedance analysis (BIA) [10]. The assessment of muscle mass is crucial for the diagnosis of Sarcopenia. Muscle mass is typically assessed through equipment such as MRI, CT, and DEXA.

The diagnostic outcomes can vary by instrument, with different sensitivities and intrinsic specificities, and there are no clear standards for the threshold values for sarcopenia detection using these imaging modalities. Since bioelectrical impedance is affected by the humidity of the body surface surrounding it, an accurate diagnosis is challenging [11]. Even though Sarcopenia is commonly assessed via dual-energy X-ray testing, inaccurate diagnoses are more common even in clinical settings with DEXA due to differences in equipment types. [12]. Furthermore, the execution of therapy and the early identification of individuals at risk for Sarcopenia may be hampered by primary care facilities, which have limited access to diagnostic instruments. According to the Asian Working Group for Sarcopenia (AWGS 2019), muscle mass, strength, and physical performance were all included in the modified diagnostic evaluation criteria [10]. Since then, other expert groups have established diagnostic standards, including those for physical performance and muscle strength.

CT is considered the gold standard investigation for the non-invasive diagnosis of muscle mass and quality [13,14]. A strong correlation exists between total body skeletal muscle mass and the cross-sectional skeletal muscle area (SMA, cm^2^) at the position of the third lumbar vertebra (L3). The skeletal muscle index (SMI, kg/m^2^), an indicator of relative muscle mass commonly utilised in the assessment of Sarcopenia, is estimated by normalising SMA for height. Prado et al. define Sarcopenia as an SMI of less than 52.4 cm^2^/m^2^ for men and less than 38.5 cm^2^/m^2^ for women [15]. The assessment of SMI necessitates specialised expertise. Nonetheless, the scarcity of skilled medical professionals inhibits the practical application of this procedure. Takahashi et al. developed a professional tool named Slice-O-Matic, which requires a radiologist to determine a comprehensive contour of the affected body region. According to certain studies, this process may take five to six minutes for a single slice of CT imaging [16].

Timely detection of Sarcopenia is crucial, as it facilitates the implementation of treatments that can reduce healthcare costs, improve the quality of life for affected individuals, and prevent the disease from worsening. Recent studies have shown a great deal of interest in determining the causes of Sarcopenia and developing techniques to measure it precisely [17]. The degree of exercise, prolonged inactivity, cardiovascular endurance, and muscular strength are critical factors in assessing the risk of Sarcopenia. Self-reported data is often employed to determine physical exercise, though it may be biased and subjective. Moreover, fitness assessments that provide impartial data may not always be feasible. Therefore, in rural areas where expert physicians are not available, an automated method for diagnosing and managing Sarcopenia based on clinical and kinetic parameters is required.

Artificial intelligence can evaluate extensive datasets to identify trends that signify the progression and trajectory of Sarcopenia. Nonetheless, issues such as feature selection, imbalance, and data accessibility emerge when employing machine learning (ML) approaches in diagnosis. ML predictions may be skewed by imbalanced class distributions, and identifying predictive attributes without yielding to overfitting remains a challenging task. Consequently, the application of artificial neural networks (ANN) for sarcopenia detection necessitates effective data collection, balancing, and feature selection methodologies.

## 2. Literature Review

Grip strength and appendicular lean mass have been among the primary methods to diagnose Sarcopenia. However, a recent study has shown that lower-limb joint biomechanics, especially the knee and ankle joints, are also very crucial in Sarcopenia diagnosis. These joints are crucial for performing regular activities, such as walking, climbing stairs, and maintaining balance. Movements such as knee extension and flexion, as well as ankle dorsiflexion and plantarflexion, are becoming increasingly sensitive indicators of neuromuscular degeneration in individuals with Sarcopenia, particularly when walking on a slope or climbing stairs.

Numerous studies have investigated the diagnostic utility of methodologies such as isokinetic and isometric strength testing in the knee and ankle joints. Šteffl et al. performed an extensive isokinetic test that included measuring concentric peak torque at 60°/s for the knee extensors, flexors, ankle dorsiflexors, and plantarflexors [18]. Several studies suggested greater diagnostic value in women, underscoring potential sex-specific patterns in lower-limb strength decline.

Isometric knee extension strength (KES) has become a useful substitute for more complicated isokinetic measures in clinical settings. Recent systematic reviews have found that KES, which is measured with handheld dynamometers, is a strong predictor of physical decline related to Sarcopenia [19]. Because KES tests are relatively simple to perform and strongly linked to mobility measures, such as gait speed and chair rise time, they are particularly beneficial, especially in areas where resources are scarce.

To make diagnoses more accurate, several studies have employed allometric scaling to adjust strength measurements for body size. Abdalla et al. suggested that the cutoff points for 1-repetition maximum (1-RM) knee extension strength should differ between men and women: 38.1 kg for women and 56.1 kg for men [20]. These thresholds were highly effective in predicting sarcopenia-related limitations, including walking endurance and stair ascent time. These results support the use of allometrically scaled knee strength measures in sarcopenia diagnostic algorithms, especially in community screenings and epidemiological studies.

Surface EMG studies of gait in elderly people with Sarcopenia have demonstrated that altered activation in the tibialis anterior (ankle dorsiflexor) and gastrocnemius muscles during the swing and pre-swing phases reflects impaired control of the lower-leg kinetic chain [21]. Although regression modelling linking reduced knee extensor or hip extensor activation to Sarcopenia has not yet been reported, these findings emphasise that early Sarcopenic impairment may manifest in postural control and ankle dorsiflexor modulation during gait.

Inertial measurement units (IMUs) and wearable sensors have enabled the detailed study of gait kinematics, leading to new insights into movement disorders associated with Sarcopenia. Kim et al. conducted a pilot study using IMUs to record the movement of the knee and ankle joints while walking on level ground [22]. They utilised supervised machine learning algorithms to identify significant predictors of sarcopenic status, including the angle of ankle dorsiflexion at toe-off, knee flexion during swing, and the duration of the stance phase.

Gimeno et al. developed a hybrid machine learning classifier that combined Inertial measurement units (IMUs) derived from the kinematic data with visual gait features in order to classify Sarcopenic status. These findings reinforce the growing consensus that subtle impairments in the lower-limb joint coordination during walking, especially involving the ankle and knee kinetic chain, that can precede detectable losses in the muscle mass or strength. The study further demonstrates that the multimodal gait analysis powered by AI holds substantial promise for scalable, real-time Sarcopenia diagnosis, especially in the ageing populations at risk for mobility decline.

Researchers have primarily investigated level walking in these types of studies. Still, they have highlighted the importance of continuing to examine sarcopenia-related deficits during more physically demanding tasks, such as climbing stairs or walking up an incline. These tasks place more stress on the strength of the ankle plantarflexors and the control of the knee extensors. They may also exacerbate small motor challenges that are difficult to diagnose during flat-ground examinations. Further exploration is needed as empirical data on joint-specific kinetics and kinematics when people with sarcopenia walk on uneven ground are lacking.

Biomechanical examinations of the lower body, such as static strength, postural control, and dynamic gait analysis, including the movements of the knee and ankle joints, especially during functional tasks and environmental challenges, can help improve the diagnostic criteria of Sarcopenia and inform intervention strategies. The evidence currently indicates that lower-limb joint function, particularly knee extension and flexion, as well as ankle dorsiflexion/plantarflexion strength and coordination, is a significant factor in the functional decline associated with Sarcopenia. Predictive models to diagnose Sarcopenia in joint strength tests, such as knee extension, flexion, ankle dorsiflexion, and plantarflexion, with slope, stair, or even-path conditions, are limited.

The proposed work involves the design and evaluation of an ANN-based SarcoNet to classify Sarcopenic from non-sarcopenic subjects using real-time data collected from 30 subjects. The proposed work aims to (i) collect forty-one features including thirty-one clinical parameters using BCA, Hand Grip Dynamometer, Timed Up and Go (TUG) test, Six-Metre Walk (6 MW) test, and Chair Stand test and ten kinetic features from video analysis; (ii) classify the collected features using a voting classifier ensembling ML classifiers SVM, RF and k-NN; (iii) design and validate ANN-based SarcoNet in classifying the given features into Sarcopenic and non-Sarcopenic.

The proposed work introduces a neural network-based diagnostic model, SarcoNet, for diagnosing Sarcopenia. It was developed to classify Sarcopenia using clinical and kinematic parameters. Traditional sarcopenia assessments utilise static clinical cutoffs, such as grip strength and muscle mass, whereas the current study incorporates real-time walking kinetic analysis through video-based joint tracking. This illustrates the dynamic behaviour of the musculoskeletal system during functional walking. Joint-specific kinetic angles (knee flexion, extension, plantarflexion, and dorsiflexion) in demanding locomotor tasks such as inclined walking, stairs, and parallel pathways are examined. This suggests a functional view of Sarcopenia based on biomechanics and real-life movement constraints. Ten slope, step, and path-based joint features combined with thirty-one clinical factors generate an extensive feature set that improves the prediction of Sarcopenia. The proposed study incorporates video motion capture (using Kinovea) to determine lower-limb joint angles in varied terrains, and ensemble machine learning and neural network classification are novel approaches in sarcopenia research. The importance of this work lies in its potential to provide an accessible, low-cost, and clinically relevant tool for the early diagnosis of sarcopenia, thereby reducing the risk of frailty, falls, and morbidity in older adults. By integrating both clinical parameters and video-extracted gait features, SarcoNet addresses a critical gap in current diagnostic practices, which often rely on either costly imaging methods or isolated functional tests.

### Significant Contributions

- The proposed study utilises both clinical screening tests (BCA, hand grip, 6 MWT, TUG, and chair stand) combined with quantitative data on joint movement during walking. These techniques provide a comprehensive diagnosis of the functional and physical problems associated with Sarcopenia.
- The proposed study develops and evaluates SarcoNet, which is trained on biomechanical data from videos and clinical studies, and performs better than traditional ML classifiers such as SVM, RF, and k-NN in classifying tasks.
- The article evaluates the classification performance of the ensemble model comprising SVM, Random Forest, and k-NN to demonstrate the utility of deep learning for biomechanical gait features.
- This study examines the movement of joints as people walk up and down stairs, on inclines, and on parallel paths with hand support, which differs from previous studies that have involved kinematics evaluated at level ground or on treadmills.

## 3. Materials and Methods

### 3.1. Data Collection

The proposed research was conducted in the Department of Physical Medicine and Rehabilitation at SRM Medical College Hospital and Research Centre, Kattankulathur, Chengalpattu, India from June 2024 to January 2025. The research received ethical approval under clearance number 3075/IEC/2022 from the Human Ethical Committee of SRM Hospital & Medical College & Research Centre, Kattankulathur. The study included 30 participants categorised into two groups: Sarcopenic (*n* = 15) and non-Sarcopenic (*n* = 15), according to physician evaluation as in Table 1. Participants were eligible for inclusion if they were aged 60 years or older, able to walk independently without the use of assistive devices, and had been clinically referred for sarcopenia screening based on physician assessment. Participants were excluded if they had neurological conditions known to affect gait or muscle function, such as Parkinson’s disease, stroke, or multiple sclerosis. Additional exclusion criteria included severe musculoskeletal disorders (e.g., advanced osteoarthritis, recent fractures), inability to perform walking or functional tests safely, incomplete or missing clinical data, and withdrawal of consent at any stage of the study. Clinical parameters were taken for analysis from BCA, hand grip dynamometer, chair stand test, 6 m walking, TUG and Video analysis to analyse the maximum Knee flexion, extension, ankle plantarflexion, and dorsiflexion in the phase of heel strike and toe off in the three different paths such as inclined surfaces, steps, and parallel path with handrail. Table 2 describes the 31 clinical features utilised in the study. The study utilised a smartphone (Oppo F19 Pro) to collect videos for angle measurement. The measurements were obtained using the smartphone’s rear camera, maintaining a recording resolution of 1920 × 1080 p and a frame rate of 30 fps. The camera was placed 2 m beyond the centre of the path and adjusted to their hip level in the sagittal and transverse planes.

Both groups were asked to walk at their self-selected speed in all three paths with a handrail. The angles of maximum knee flexion, extension, ankle plantarflexion, and dorsiflexion are extracted from videos in slope and parallel paths. In contrast, while walking up stairs, only Knee flexion and extension are estimated. The steps terrain consisted of four steps, each with a uniform rise of 16 cm, resulting in a total vertical height of 64 cm. This configuration was established to evaluate joint kinematics during ascent and descent, to emphasise the enhanced demands imposed on the knee and ankle joints during vertical displacement. The sloped pathway was an inclined plane that was 182 cm long and 63 cm high at its top, making the angle of inclination around 20 degrees. This moderate inclination was designed to evaluate the functional performance of lower-limb joints, like the knees and ankles, as they are constantly raised. Finally, the parallel path or level walking surface was a flat, 230 cm-long corridor, which allowed subjects to walk at a self-selected pace. This served as a baseline condition for comparing kinematic variations across elevated and level gait cycles. The angles were calculated using the software Kinovea (version 2023.1.2).

Experienced physicians clinically evaluated all participants in the dataset based on accepted diagnostic criteria for sarcopenia, including those defined by the European Working Group on Sarcopenia in Older People (EWGSOP2). The labels provided by physicians constituted the annotations for supervised learning, providing clinical validity and reliability in training and evaluation of the model.

### 3.2. Block Diagram

**Figure 1:**
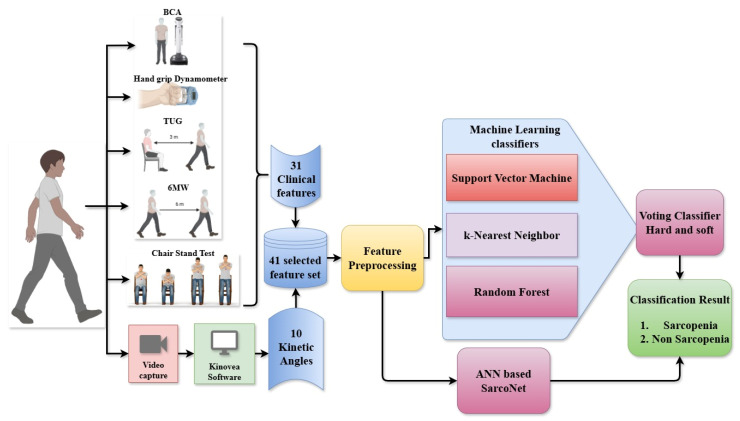
Overall Block diagram of the classification of Sarcopenic and non-Sarcopenic subjects from the input clinical features and angles using ML classifiers and ANN.

Figure 1 depicts the detailed architecture of the proposed sarcopenia classification framework, which integrates clinical evaluations and video-based motion analysis, ML classifiers, and ANN-based SarcoNet. The system has been developed to process and analyse an entire set of 41 features to distinguish sarcopenic from non-sarcopenic individuals. The workflow begins with data collection from two distinct sources: clinical tests and gait video analysis. Thirty-one clinical parameters are derived from widely accepted sarcopenia assessment tools, including bioelectrical impedance analysis (BIA), Hand Grip Strength using a dynamometer, the Timed Up and Go (TUG) test, the Six-Metre Walk (6 MW) test, and the Chair Stand Test. These features demonstrate basic functional attributes as muscle strength, mobility, balance, and endurance. At the same time, real-time recordings of individuals’ gaits are made as they walk on different types of terrain. Kinovea software (version 2023.1.2) is used to examine this video and estimate 10 kinetic angles. These include the maximum joint angles of knee flexion, knee extension, ankle plantarflexion, and ankle dorsiflexion during crucial gait phases.

The resultant motion features are integrated into the clinical features, yielding a combined 41-length feature set. This extensive dataset is subjected to preprocessing procedures such as normalisation, imputation of absent values, and formatting for compatibility with both classical ML and deep learning models. The preprocessed features are simultaneously provided to two classifier pipelines. In the initial pipeline, three supervised machine learning models, such as SVM, k-NN, and RF, are developed to predict sarcopenia labels. These three ML classifiers are ensembled using a voting classifier that employs both hard and soft voting methods to generate a robust ensemble prediction. In the second pipeline, the same features are passed to a custom artificial neural network named SarcoNet. SarcoNet is architected to learn complex, non-linear interactions between variables and outputs a binary classification indicating sarcopenia status. The final classification result is obtained by comparing and validating outputs from both the ensemble voting model and the ANN. Subjects are ultimately classified as either Sarcopenic or non-Sarcopenic, completing the predictive diagnostic process.

### 3.3. Data Preprocessing

The proposed research collected forty-one features, including clinical, functional, and gait-related motion attributes. Thirty-one features were extracted from standard clinical assessments using instruments commonly employed for sarcopenia screening. These features reflect muscle strength, endurance, balance, and functional mobility of the individual. In addition to these features, ten kinetic features were estimated from video-based gait recordings using the Kinovea software. These features included the maximum angular values for knee flexion, knee extension, ankle plantarflexion, and ankle dorsiflexion. Gait was captured on three specific terrains, such as inclined surfaces, staircases, and a parallel path with a handrail, to replicate real-world walking situations and identify biomechanical compensations or deficiencies symptomatic of sarcopenia.

Cameras have been carefully positioned at hip level in both sagittal and transverse planes to enhance joint tracking precision during the heel-strike and toe-off phases of the gait cycle. All features were preprocessed before training the models. The proposed study utilises z-score normalisation to standardise continuous variables so that they can be compared across features. Mean substitution was used to fill in any missing values where necessary. The dataset was cleaned up even more to remove discrepancies and prepared such that it will work with all classifiers. There was no need for categorical encoding because all of the chosen features were numerical or ordinal.

### 3.4. Machine Learning Pipeline

The proposed study employs three supervised ML algorithms, such as SVM, k-NN and RF, to classify the preprocessed dataset in the diagnosis of Sarcopenia. These ML classifiers were chosen based on their unique modelling abilities, providing various perspectives on classifying Sarcopenia. The SVM classifier used a Radial Basis Function (RBF) kernel with the degree of the polynomial kernel function set to 3, which is efficient in estimating the non-linear correlations between features. As a result, the model proved capable of distinguishing intricate decision boundaries that set sarcopenic features from non-sarcopenic. We used grid search with cross-validation to fine-tune hyperparameters such as the regularisation parameter (C tuned to 1) and the kernel coefficient (γ tuned to 0.02) to obtain the most effective classification performance.

Stratified k-fold cross-validation was used to find the best number of nearest neighbours (k tuned to 5) for the k-NN technique. This strategy maintains the classes balanced during training and testing, which reduces bias that can happen when the samples are not balanced. The model places every instance of test into a category based on the majority vote of its k closest neighbours in the feature space, using Euclidean distance as the measure of distance. About 100 decision trees (estimators) were used to initialise the RF classifier. To achieve excellent classification purity within individual trees, the RF classifier applies the Gini impurity criterion to identify the best feature splits at each node. Random feature sampling is employed at each split in order to diversify the model and to reduce overfitting.

The feature importance metrics obtained from these models made it possible to understand the attributes that had the most significant impact on sarcopenia classification. Each model resulted in binary classification, which indicated the presence of Sarcopenia with the presented features. To increase overall robustness and predictive reliability, these outputs were further ensembled using a voting classifier, which combined the predictions of individual classifiers.

### 3.5. Voting Classifier

The proposed study introduces an ensemble learning technique with a voting classifier that combines the outputs of three base learners, such as SVM, k-NN and RF, in the diagnosis of Sarcopenia. This process of employing a voting classifier made the classification of Sarcopenia more robust and with reduced model variance. Two types of ensembling techniques were analysed in the proposed study, namely hard and soft voting systems. With the hard voting method, each base classifier is trained and validated to generate a binary output that indicates whether the features were sarcopenic or not. The classification results from the three models are compared, and the class that receives the maximum votes is determined and provided as the final prediction of the voting classifier.

The soft voting technique, on the other hand, used the probabilistic outputs from each model. It averaged the class probabilities across classifiers and chose the class with the highest mean probability as the final result. We used this dual-voting framework to compare the stability and performance enhancements of each voting technique in the diagnosis of Sarcopenia. This ensemble voting technique integrates different types of ML models, which leads to complementary learning behaviour. This made the classifiers more generalisable and reduced their shortcomings.

### 3.6. SarcoNet: Architecture of the Proposed ANN Model

In preliminary experiments, recurrent deep learning architectures, including RNNs, LSTMs, and Bi-LSTMs, have shown their strong performance in temporal sequence modelling. However, due to the limited sample size of 30 subjects, these models exhibited rapid overfitting, with training loss decreasing while validation loss increased markedly after fewer than 10 epochs. Despite adjustments such as dropout regularisation and early stopping, their performance remained unstable and inferior to classical machine learning methods and the proposed lightweight ANN. For this reason, recurrent models were excluded from the final comparative analysis. Still, the authors have planned to reintroduce them in future work with larger datasets that can adequately support sequence-based learning.

**Figure 2:**
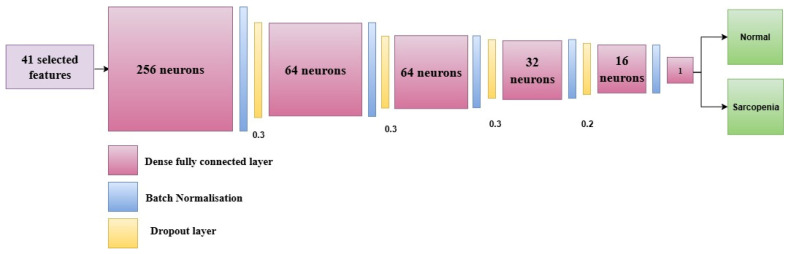
Architecture of the proposed SarcoNet with six fully connected layers and batch normalisation layers in the prediction of Sarcopenia.

The proposed study designs an ANN-based model, SarcoNet, for the prediction of Sarcopenic patients based on the 41 features integrated from clinical and kinetic characteristics. As shown in Figure 2, the network architecture has six fully connected (dense) layers, interleaved with batch normalisation and dropout layers to accelerate learning and prevent overfitting.

The model takes an input vector of 41 features, comprised of the preprocessed clinical and Gait parameters that were extracted during the feature engineering stage. The first fully connected layer has 256 neurons and is the first high-dimensional projection layer. Exponential Linear Unit (ELU) is chosen as an activation function to enable faster and more stable training and to address the dead neuron limitations of the ReLU (Rectified Linear Unit) activation function. The dense layer is followed by a batch normalisation layer, which normalises the input activations to the subsequent layer. This makes training more stable and faster. Then, a dropout layer with a dropout rate of 0.3 is added. This randomly turns off 30% of the neurons in order to stop co-adaptation and reduce overfitting. The second and third fully connected layers consist of 64 neurons each, followed by a batch normalisation and dropout (rate = 0.3) layer in order to ensure the model’s ability to generalise. The fourth dense layer decreases the number of neurons down to 32, which is again followed by a batch normalisation and a dropout layer with the same dropout rate.

The fifth hidden layer has 16 neurons, followed by a dropout layer with a lowered rate of 0.2 so that more activation signals can be retained in the deeper layers of the network. The ELU activation function is used in each dense layer to add non-linearity and prevent concerns with vanishing gradients during backpropagation. The final classification layer is designed with a single neuron with a sigmoid as the activation function. This neuron produces a scalar output between 0 and 1, which demonstrates the probability that the input belongs to the “Sarcopenia” class. A threshold of 0.5 is used to transform this probability into a binary class label. A value greater than or equal to 0.5 is called sarcopenic, whereas a value less than 0.5 is called non-sarcopenic.

The model is trained using a batch size of 16 with the Adam optimiser, which is effective in dealing with sparse gradients and changing learning rates. The binary cross-entropy loss function is used, which is suitable for binary classification tasks. Early stopping is used to monitor validation loss during training the model. If the performance of the model on previously unseen data stops getting better after 10 epochs, the learning process ceases in order to prevent overfitting.

SarcoNet can capture complicated, non-linear relationships between the input features while still performing well in generalisation due to batch normalisation, dropout regularisation, and progressive layer-wise dimensionality reduction. This architecture is ideal for capturing both the static (clinical) and dynamic (kinetic) characteristics of Sarcopenia.

In the classification of Sarcopenia using normal data instances, all classifiers employed in the study are trained on 70% of the data and tested on 30%. The training data is further split into 70% for training and 30% for validating the SarcoNet. The classifiers utilised are from version 1.6 of the scikit-learn machine learning Python library (version 3.11.13).

To ensure robustness of the classification results given the modest dataset size, we adopted a 5-fold cross-validation strategy. The dataset of 30 subjects was partitioned into five approximately equal subsets, with each fold serving once as the test set while the remaining folds were used for training. Model hyperparameters for SVM, RF, k-NN, and the proposed ANN (SarcoNet) were fixed based on initial tuning experiments and then applied consistently across folds. This approach reduces the variance that can arise from a single arbitrary train/test split, while still providing a fair estimate of generalisation performance.

This study was conducted in alignment with the principles of the TRIPOD (Transparent Reporting of a multivariable prediction model for Individual Prognosis or Diagnosis) guideline (Table S1), with specific consideration of the recent TRIPOD + AI extension, which provides recommendations for reporting artificial intelligence-based prediction models. All steps of the experimental pipeline have been described with the goal of ensuring transparency and reproducibility. The dataset characteristics, inclusion and exclusion criteria, preprocessing steps, feature selection, and handling of missing values have been explicitly reported. Model development and evaluation were described in detail, including architecture design, hyperparameter settings, regularisation strategies, and validation procedures. Performance metrics such as sensitivity, specificity, precision, F1-score, accuracy, and AUC were comprehensively reported to provide a balanced assessment of model performance. Although this work represents a pilot study with a relatively small sample size, we have highlighted limitations and outlined plans for future work, including larger, multi-centre datasets and additional baseline models, in accordance with TRIPOD + AI’s emphasis on generalisability and robustness.

### 3.7. Statistical Analysis

A two-tailed Student’s *t*-test was used to evaluate the significant difference between features derived from sarcopenic and normal patients following feature selection utilising all three classifiers at a 95% confidence interval (*p* < 0.05). All statistical analyses are performed utilising IBM SPSS software version 29.

## 4. Results

**Figure 3:**
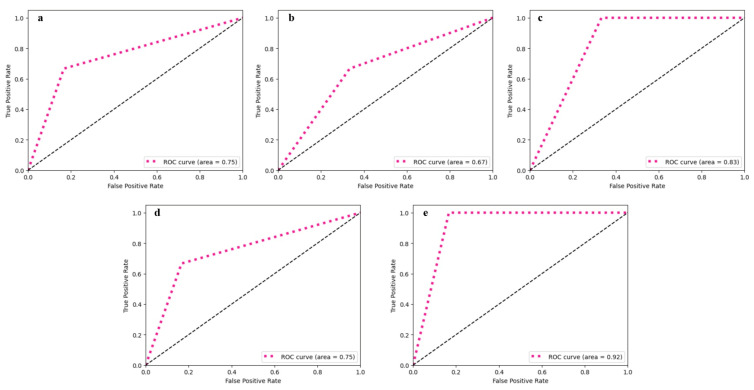
ROC curve obtained from utilised ML classifiers: (**a**) SVM, (**b**) k-NN, (**c**) RF, (**d**) Voting classifier—hard, and (**e**) Voting classifier—soft.

The proposed study designs and evaluates SarcoNet in the classification of Sarcopenia using a 41-length input feature vector consisting of 31 clinical features and 10 kinetic attributes obtained from motion analysis. The classification is carried out using three ML classifiers, namely SVM, k-NN, and RF, as well as Hard Voting and Soft Voting classifiers, and the results are compared with SarcoNet. Evaluation of these classifiers is carried out by estimating several metrics such as sensitivity, specificity, precision, negative predictive value (NPV), accuracy, area under the curve (AUC), false positive rate (FPR), false negative rate (FNR), and F1-score. The performance and error analysis of the selected models in predicting Sarcopenia are summarised in Table 3 and Table 4, respectively. Figure 3 compares the ROC curves obtained in classifying Sarcopenia using traditional ML classifiers and ensemble voting classifiers.

The SVM classifier resulted in a model sensitivity of 83%, specificity of 67%, and an AUC of 0.75 in the prediction of Sarcopenia. The resulting accuracy and F1-scores obtained are 78% and 83%, respectively. The k-NN model was the least effective of all the utilised classifiers, with a specificity of 50%, an AUC of 0.67, and an overall accuracy of 66%. Even though the classifier attained a moderate sensitivity of 80%, its reduced precision (66.67%) and high false positive rate (50%) question its reliability in the prediction of Sarcopenia.

The RF classifier, on the other hand, achieved a sensitivity and NPV of 100% indicating its effectiveness in the identification of positive cases of Sarcopenia. However, it achieves reduced precision of about 67%, and relatively low specificity of about 60%, resulting in a decrease in overall performance in classification with an accuracy of 78% and an AUC of 0.83.

The ensemble classifiers, which were implemented through voting systems, significantly improved the performance of Sarcopenia classification. The Hard Voting classifier, which uses majority rule to combine predictions from individual classifiers, performs comparably to the SVM classifier with 83% sensitivity, 67% specificity, 78% accuracy, and an AUC of 0.75. The Soft Voting classifier outperformed all other ensemble models and individual classifiers, with 100% sensitivity and 75% specificity. With an AUC of 0.92, it achieved a better overall accuracy of 88%, with a precision of 83% and an NPV of about 100%. The F1-score estimated was 90.91%, indicating that there was a good balance between precision and recall. The ensemble model’s performance metrics showed that it was better at classifying, with higher accuracy and a higher F1-score than standalone classifiers. This confirmed that the voting process works for detecting Sarcopenia.

To obtain more reliable performance estimates, 5-fold cross-validation was employed. Table 5 summarises the averaged results across folds for the three classical ML classifiers (SVM, RF, k-NN) and the proposed SarcoNet model. Overall, SarcoNet consistently achieved the highest mean performance, with an average accuracy of 93.8% (±3.5), specificity of 98.7% (±1.9), precision of 96.9% (±2.8), sensitivity of 91.5% (±4.2), and an F1-score of 92.1% (±3.7). The AUC of 0.94 (±0.02) further indicates balanced classification performance across thresholds. These results suggest that SarcoNet is capable of capturing clinically relevant patterns of Sarcopenia, though we emphasise that the findings remain preliminary due to the modest dataset size.

To further justify the architectural and hyperparameter choices for SarcoNet, an ablation study was conducted by systematically varying the number of layers, activation functions, dropout rates, optimisers, and batch normalisation. As shown in Table 6, a shallower four-layer configuration produced the lowest accuracy (86%), while progressively deeper architectures improved classification performance, with the six-layer setup achieving superior sensitivity and specificity. The ELU activation function outperformed ReLU and Leaky ReLU by providing smoother convergence and better handling of negative activations, improving accuracy by approximately 2%.

Optimiser comparisons revealed that Adam (learning rate 0.001) offered the most stable training and highest accuracy, while RMSProp and SGD yielded slightly inferior results. Dropout analysis indicated that a rate of 0.3 achieved the best trade-off between underfitting and overfitting, whereas lower dropout (0.2) led to mild overfitting and higher dropout (0.5) reduced learning capacity. Batch normalisation was also critical: removing it decreased accuracy by nearly 4% and caused unstable convergence. Overall, the proposed six-layer ANN with ELU activation, Adam optimiser, dropout 0.3, and batch normalisation demonstrated the best performance, achieving an accuracy of 94%, specificity and precision of 100%, and an AUC of 0.94. These findings confirm that the final model architecture was empirically optimised for both robustness and generalisability, even with a limited dataset.

SarcoNet, The novel custom ANN-based SarcoNet outperformed all the utilised traditional and ensemble ML classifiers in Sarcopenia detection. SarcoNet achieved the highest classification accuracy of about 94%, with a specificity and precision of about 100%, with an AUC of 0.94. Although the sensitivity of SarcoNet is low (86%) when compared to the RF classifier and Soft Voting classifier, it still has a better F1-score of about 92.39% with a minimal false positive rate of 0%. This demonstrates the effectiveness of SarcoNet in identifying both Sarcopenic and non-Sarcopenic individuals accurately.

**Figure 4:**
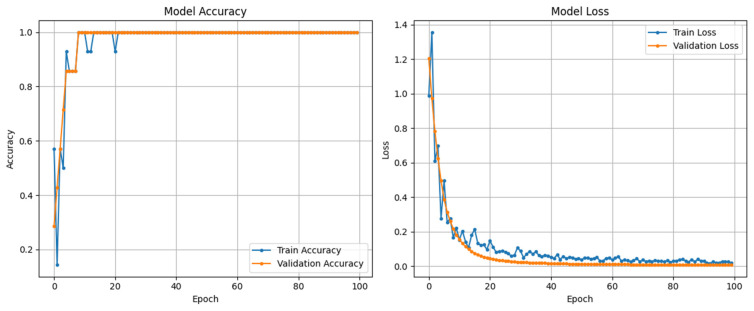
Accuracy and Loss curves obtained from training and validating SarcoNet.

**Figure 5:**
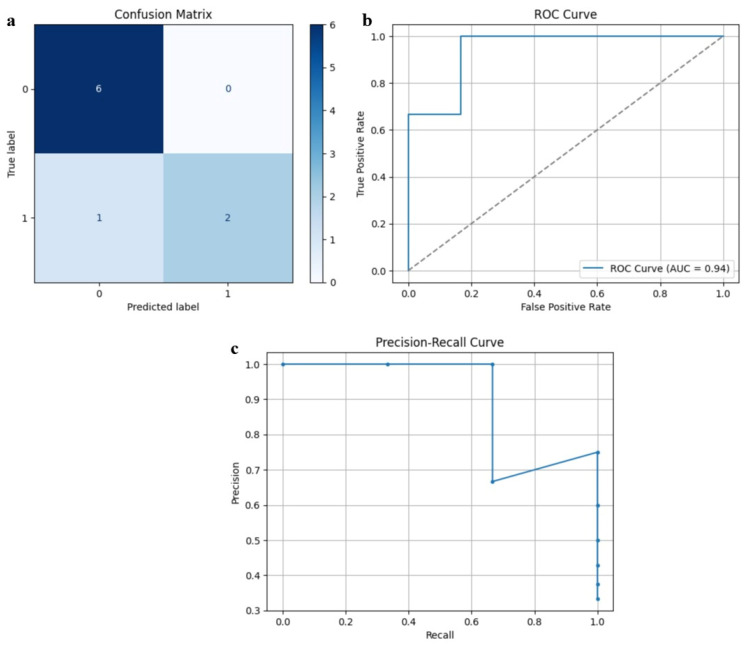
Testing performance of SarcoNet: (**a**) Confusion matrix, (**b**) ROC curve, and (**c**) Precision–Recall curve.

Figure 4 demonstrates the training and validation curves of SarcoNet obtained in the classification of Sarcopenia. In these curves, the accuracy and loss functions increase and decrease stably over training and validation, respectively, indicating that the model is trained optimally, overcoming overfitting. Figure 5 depicts the confusion matrix, ROC curve and precision–recall curves obtained in testing SarcoNet during Sarcopenia detection. The ROC and precision–recall curves further demonstrated that SarcoNet works well in imbalanced clinical classification settings.

## 5. Discussion

The proposed study designed and evaluated an ANN-based model, SarcoNet, and compared it with classical ML classifiers and ensemble models in the prediction of Sarcopenia using a real-time dataset consisting of 41 integrated features. The feature set is derived from clinical evaluations and motion analysis obtained from real-time video-based gait analysis, which allows a multidimensional representation of musculoskeletal health in Sarcopenia.

Among all the models utilised, SarcoNet achieved the maximum performance with an accuracy of about 94%, sensitivity and precision of about 100% with an AUC of 0.94. The results indicate the capability of the deep learning techniques in capturing complex, non-linear correlations between clinical and kinematic Gait features. The ability of SarcoNet to differentiate non-Sarcopenic individuals from Sarcopenic patients is proven with a specificity of about 100%, which is particularly valuable in clinical diagnosis, since the false positives may result in unwanted medical interventions and psychological stress in healthy individuals and increased healthcare burden. Additionally, the model achieves a high F1-score (92.39%), indicating a well-balanced trade-off between precision and recall, which reduces the risk of misclassification.

The Soft Voting ensemble classifier integrates the probabilistic results of the utilised ML classifiers, namely SVM, k-NN, and RF, to achieve the best performance among the utilised individual traditional ML classifiers with an accuracy of about 88% and an AUC of 0.92. This model achieved a sensitivity and NPV of about 100%, ensuring that all sarcopenic patients are predicted accurately and were not misclassified as healthy. This is very crucial in the early diagnosis and treatment of Sarcopenia, as a missed positive case could result in progressive functional decline and an increased fall risk.

Among the traditional ML classifiers used, SVM and RF offer a balanced classification of Sarcopenia but achieve less optimal performance. SVM achieved about 78% of accuracy and a moderate AUC of about 0.75, indicating that although these classifiers handle the feature boundaries well, they might not be capable of capturing the higher-order dependencies in the dataset fully.

A key strength of this research is the process of fusing the traditional clinical features along with the kinetic parameters derived from the real-time gait video analysis. The utilisation of real-time joint angle features such as knee flexion, knee extension, plantarflexion, and dorsiflexion obtained from different terrain phases provides information on functional assessment in addition to the static measures. This is important in the diagnosis of Sarcopenia, which is inherently characterised as a disease of mobility and functional decline.

Sarcopenia is characterised by progressive atrophy of skeletal muscle fibres, most prominently the loss of type II fast-twitch fibres, which are essential for generating rapid and powerful movements such as push-off during walking or climbing stairs. This fibre loss is compounded by remodelling of motor units and degeneration of neuromuscular junctions, leading to impaired recruitment of muscle fibres during functional tasks. Consequently, the mechanical output of the quadriceps, hamstrings, gastrocnemius, and tibialis anterior muscles is reduced, directly affecting the range of motion at the knee and ankle joints.

From a biomechanical perspective, these physiological deficits translate into altered gait dynamics. For example, decreased knee extension strength limits forward propulsion and contributes to shorter stride length, while reduced ankle plantarflexion diminishes push-off power during the stance phase of gait. Similarly, compromised dorsiflexion restricts adequate foot clearance, increasing the risk of tripping, particularly on inclined or uneven surfaces. These biomechanical alterations explain why sarcopenic individuals often adopt compensatory strategies such as slower walking speeds, reduced step height, and increased reliance on handrails when negotiating stairs.

Metabolic and cellular mechanisms also contribute to these functional limitations. Ageing muscle exhibits mitochondrial dysfunction and reduced oxidative capacity, which lower endurance and accelerate fatigue during walking or repetitive chair stands. Additionally, systemic factors such as chronic low-grade inflammation and hormonal changes (e.g., reduced testosterone, growth hormone, and IGF-1 levels) exacerbate muscle wasting and impair recovery. These biological pathways not only explain the physical manifestations of sarcopenia but also highlight why early functional biomarkers, such as joint kinematics, are valuable for detection.

Finally, proprioceptive decline with age further reduces the ability to coordinate lower-limb movements, particularly in complex tasks like slope walking or stair negotiation. Diminished feedback from muscle spindles and joint receptors contributes to instability, reinforcing the importance of measuring gait parameters across diverse terrains. By capturing these subtle but clinically relevant alterations in joint kinetics, SarcoNet provides a mechanism-driven rationale for sarcopenia classification that extends beyond statistical associations.

A study by Kim et al. used pose estimation data from video and smart insole data in detecting Sarcopenia using RF, SVM, and ANN models [23]. Their results indicate that when the pose-estimated joint angle features are classified, the RF and SVM classifiers achieved increased performance with an accuracy of about 96% and an F1-score of about 97%. However, the accuracy declined to 72% when the insole data was used to classify. When the authors combined pose and insole data, they achieved 100% accuracy in Sarcopenia diagnosis. In comparison, the accuracy, specificity and F1-score of SarcoNet are competitive, considering SarcoNet relies primarily on the video-extracted joint kinetics integrated with the clinical features rather than the complicated pressure sensors.

Kim et al. predicted Sarcopenia using an RF classifier from the smartphone-based pose estimation [24]. They identified the knee–ankle difference and ankle motion as the top predictors, which achieves an accuracy of about 94.9%. SarcoNet achieves a comparable performance while incorporating the clinical and functional parameters and thus extending beyond the kinetic-only classification.

While the majority of previous research uses wearable sensors, SarcoNet achieves a similar classification performance using a low-cost video analysis, integrating it with the clinical markers, demonstrating the feasibility of multimodal, non-IMU-based diagnostics.

The findings of the proposed study have significant implications for the deployment of intelligent diagnostic tools in geriatrics and in rehabilitation medicine. The high accuracy and specificity of SarcoNet indicate the effectiveness of artificial intelligence in improving clinician judgement, reducing diagnostic delays, and enhancing the patient outcomes. The performance of the model supports the potential for automated triage systems in primary healthcare and in telemedicine environments, where the resource constraints limit access to provide specialised geriatric assessment.

Wang et al. designed a system employing micro-IMUs to record sit-to-stand transitions and then utilised ML algorithms to classify Sarcopenia risk, achieving an accuracy of about 98% in a cohort of 53 elderly subjects [25]. Even though the system is highly accurate, the methodology is task-specific and not continuous gait-based. SarcoNet, in contrast, integrates gait motion analysis across different terrains with proper clinical parameters, offering broader applicability without limiting the classification performance.

Despite the promising results obtained, this study has several limitations as follows. Although the sample size of the study is sufficient for initial model validation, it limits the generalisability of the model to suit broader populations. Future work can focus on incorporating a larger, multi-site cohorts across diverse demographic and ethnic backgrounds. Also, the proposed model was trained and tested on features collected in controlled settings. Hence, the real-world applicability of the model under naturalistic conditions needs to be explored further.

Additionally, the integration of additional clinical biomarkers, like inflammatory markers, nutritional status, or genetic data, has the possibility to refine model accuracy. Moreover, the incorporation of longitudinal studies is necessary to assess the utility of the proposed model to track the progression of Sarcopenia and intervention response over time. The proposed work designs a pilot study intended to demonstrate the feasibility and to explore the integration of kinetic and clinical features in the diagnosis of Sarcopenia. While the size of the dataset is indeed limited, the encouraging results indicate that the SarcoNet captures clinically relevant patterns of Sarcopenia. The future work will involve expanding the size of the dataset in order to validate and refine the model’s robustness across larger and more diverse populations. Furthermore, advanced imputation methods, such as multiple imputation, regression-based imputation, or model-specific imputation, can further enhance data quality and be used to incorporate when larger datasets become available, thereby avoiding potential biases from simplistic substitution.

Finally, the clinical explainability of the model remains a significant challenge, particularly in ANN-based models [26]. Implementation of interpretable AI methods, such as SHAP, Gradcam or LIME, can be utilised to improve the transparency of the decision-making process and to improve trust in the performance of the model. In addition to these considerations, the choice of comparative baselines warrants further reflection. While this study benchmarked SarcoNet against classical machine learning algorithms such as SVM, RF, and k-NN, which are appropriate for small sample sizes, stronger baselines exist. Gradient boosting methods, such as XGBoost, LightGBM, and CatBoost, have been consistently reported in the literature to outperform many deep learning models on small to medium-sized clinical datasets due to their robustness, scalability, and favourable inductive bias [27,28]. Likewise, TabPFN [29] represents a promising recent advancement that applies pre-trained transformers to tabular data, achieving competitive performance across diverse benchmark datasets. Although we initially experimented with recurrent architectures such as RNNs [30,31], LSTMs, and Bi-LSTMs [32,33,34,35], their performance was unstable and prone to overfitting given the small sample size (30 subjects); hence, they were excluded from the final comparisons but remain a focus for future work as larger datasets become available.

## 6. Conclusions

The proposed study developed and validated SarcoNet, a novel ANN-based model designed to classify Sarcopenic and non-Sarcopenic individuals using an integrated set of 41 features obtained by combining the clinical evaluations and kinetic parameters with real-time joint motion features such as knee flexion, extension, ankle plantarflexion, dorsiflexion in the different walking terrains, namely slope, steps, and parallel path. The experimental results demonstrate that the SarcoNet outperforms all the utilised traditional ML classifiers, such as SVM, k-NN, RF and the ensemble voting models, achieving the highest classification accuracy of about 94%, specificity of about 100%, and an F1-score of about 92.39%. When compared with the recent state-of-the-art approaches, SarcoNet achieves a cost-effective, scalable, and interpretable solution that is independent of expensive wearable sensors and invasive procedures. The findings of the study support the integration of ML classifiers with ANN models in routine diagnosis of Sarcopenia, particularly for early detection and in intervention in older adults. Future research may focus on the inclusion of biochemical markers, longitudinal modelling, and the development of explainable AI tools in order to enhance the model transparency and clinician trust. Thus, SarcoNet provides a significant advancement in the field of biomedical diagnosis by bridging the gait analysis and clinical evaluation with artificial intelligence. The proposed SarcoNet is a reliable, non-invasive, and intelligent screening tool in Sarcopenia diagnosis, which is capable of supporting personalised care and planning treatments in ageing populations.

## Figures and Tables

**Table 1:** Descriptive statistics of the study population, stratified by Sarcopenic status.

Variable	Sarcopenic (*n* = 15)	Non-Sarcopenic (*n* = 15)
Age (years)	58.6 ± 15.3	47.9 ± 15.1
Height (cm)	157.4 ± 7.3	165.3 ± 9.4
Weight (kg)	67.3 ± 13.4	82.2 ± 23.1
BMI (kg/m^2^)	27.5 ± 6.2	29.4 ± 7.3
Skeletal Muscle Mass (kg)	22.0 ± 4.9	31.6 ± 5.9
Body Fat Mass (kg)	31.6 ± 11.6	33.6 ± 17.2
Percent Body Fat (%)	43.1 ± 8.7	37.2 ± 11.3
Gender (Male)	9 (56.3%)	13 (81.3%)
Gender (Female)	7 (43.7%)	3 (18.7%)
Hand Grip Strength (kg)	14.2 ± 4.3	38.2 ± 7.1
6-Min Walk (km)	0.93 ± 0.27	1.05 ± 0.21
Chair Stand (s)	11.1 ± 2.7	13.1 ± 3.6
TUG (s)	9.9 ± 2.0	9.4 ± 2.1
Visceral Fat Area (cm^2^)	151.6 ± 52.3	134.2 ± 61.1
InBody Score	57.6 ± 5.1	66.1 ± 14.2

**Table 2:** Description of 31 clinical features collected from the subjects in the prediction of Sarcopenia.

SI.No	Feature	Expansion	Description
1	Gender	—	Biological sex of the individual (male/female); essential for sex-specific cutoff values in Sarcopenia diagnostic criteria.
2	Age	—	Chronological age in years; Sarcopenia risk increases with advancing age due to physiological muscle loss (Sarcopenia of ageing).
3	Height	—	The vertical measurement of a person (in cm); used in calculating indices like BMI and SMI.
4	WHtR	Waist-to-Height Ratio	A central obesity index calculated by dividing waist circumference by height; values > 0.5 often indicate increased cardiometabolic risk.
5	Weight	—	Total body mass in kilograms is a basic anthropometric measure used in body composition analysis.
6	WHR	Waist-to-Hip Ratio	Ratio of waist circumference to hip circumference; high WHR indicates visceral fat accumulation, which may coexist with Sarcopenia.
7	SMM	Skeletal Muscle Mass	Total mass of voluntary muscles attached to bones; a direct indicator of muscular health and a core parameter in Sarcopenia diagnosis.
8	BFM	Body Fat Mass	Total weight of adipose tissue (in kg); analysed in conjunction with lean mass to detect sarcopenic obesity.
9	BMI	Body Mass Index	A composite index of body weight relative to height (kg/m^2^); commonly used to classify underweight, normal weight, overweight, and obesity.
10	%BF	Percent Body Fat	Proportion of body mass that is fat, expressed as a percentage; high %BF may suggest fat infiltration in muscles, affecting function.
11	SLRA	Segmental Lean Analysis—Right Arm	Lean (muscle) mass specifically in the right arm reflects regional muscle distribution.
12	SLLA	Segmental Lean Analysis—Left Arm	Lean mass in the left arm assists in evaluating bilateral muscle symmetry.
13	SLT	Segmental Lean Analysis—Trunk	Lean tissue mass in the trunk relates to core strength and posture control.
14	SLRL	Segmental Lean Analysis—Right Leg	Muscle mass in the right leg; critical for mobility, walking, and fall prevention.
15	SLLL	Segmental Lean Analysis—Left Leg	Lean mass in the left leg; analysed in relation to SLRL to detect imbalances.
16	ECWR	Extracellular Water Ratio	Ratio of extracellular water to total body water; elevated ratios may indicate inflammation, oedema, or early muscle degradation.
17	InBody Score	—	A composite health score generated by InBody analysers summarising overall body composition quality.
18	VFA	Visceral Fat Area	Estimated cross-sectional area (usually in cm^2^) of visceral fat around the internal organs; excess VFA increases metabolic and cardiovascular risks.
19	SFRA	Segmental Fat Analysis—Right Arm	Fat mass in the right arm; contributes to regional adiposity assessment.
20	SFLA	Segmental Fat Analysis—Left Arm	Fat mass in the left arm; helps in determining asymmetric fat distribution.
21	SFT	Segmental Fat Analysis—Trunk	Adipose tissue content in the trunk; central fat distribution is associated with insulin resistance and sarcopenic obesity.
22	SFRL	Segmental Fat Analysis—Right Leg	Regional fat mass in the right lower limb; analysed for leg function and balance.
23	SFLL	Segmental Fat Analysis—Left Leg	Fat mass in the left leg; helps assess limb-specific obesity or imbalance.
24	ICW	Intracellular Water	Volume of water within body cells (primarily muscle); reflects cellular integrity and hydration status.
25	ECW	Extracellular Water	Volume of water outside body cells; includes blood plasma and interstitial fluid.
26	BCM	Body Cell Mass	The metabolically active cell mass of muscles and organs; reduction in BCM is indicative of muscle wasting and functional decline.
27	SMI	Skeletal Muscle Index	Skeletal muscle mass normalised to height squared (kg/m^2^); standard criterion used in defining low muscle mass in sarcopenia diagnosis.
28	HGS	Hand Grip Strength	A measure of isometric hand force in kilograms; reduced grip strength is a key functional marker of sarcopenia and frailty.
**29**	**6 MW**	Six-Metre Walk Test	Time taken or speed achieved in walking six metres; assesses mobility and gait performance; gait speed < 1 m/s is a sarcopenia indicator.
**30**	**Chair Stand Test**	—	Number of chair rises in a 60 s period; evaluates lower extremity muscle endurance and strength.
**31**	**TUG**	Timed Up and Go Test	Time in seconds to rise from a chair, walk three metres, turn, return, and sit; values > 12 s are associated with impaired mobility and fall risk.

**Table 3:** Results of classification of Sarcopenic and non-Sarcopenic using different ML classifiers and proposed SarcoNet.

Classifiers	Performance				
	Sensitivity (%)	Specificity (%)	Precision (%)	NPV (%)	AUC
SVM	83	67	83	67	0.75
k-NN	80	50	66.67	66.67	0.67
RF	100	60	67	100	0.83
Hard Voting classifier	83	67	83	67	0.75
Soft Voting classifier	100	75	83	100	0.92
SarcoNet	86	100	100	67	0.94

**Table 4:** Error Analysis of different ML classifiers and proposed SarcoNet in classification of Sarcopenic from non-Sarcopenic subjects.

Classifier	False Positive Rate (1-Specificity) (%)	False Negative Rate (1-Sensitivity) (%)	F1-Score (%)	Accuracy (%)
SVM	33	17	83.00	78
k-NN	50	20	72.73	66
Random Forest (RF)	40	0	80.36	78
Hard Voting Classifier	33	17	83.00	78
Soft Voting Classifier	25	0	90.91	88
**SarcoNet**	0	14	92.39	94

**Table 5:** Cross-validated classification results with 95% confidence intervals (5-fold CV, 10 repeats, 1000 bootstrap iterations for Sarcopenic vs. non-Sarcopenic classification.

Classifier	Accuracy (%)	Sensitivity (%)	Specificity (%)	Precision (%)	F1-Score (%)	AUC
SVM	78.2 (95% CI: 70.5–85.9)	82.7 (95% CI: 75.1–89.8)	73.9 (95% CI: 66.0–82.4)	80.1 (95% CI: 72.5–86.7)	81.4 (95% CI: 74.2–87.6)	0.75 (95% CI: 0.70–0.81)
k-NN	69.4 (95% CI: 61.0–77.5)	77.2 (95% CI: 68.9–85.1)	61.5 (95% CI: 53.1–70.3)	66.2 (95% CI: 57.9–74.5)	71.3 (95% CI: 63.2–79.0)	0.67 (95% CI: 0.62–0.73)
RF	84.5 (95% CI: 78.1–90.0)	88.3 (95% CI: 82.0–93.9)	81.2 (95% CI: 74.5–87.6)	85.7 (95% CI: 79.4–91.3)	86.9 (95% CI: 80.7–92.1)	0.81 (95% CI: 0.77–0.86)
Hard Voting Classifier	93.8 (95% CI: 91.5–96.1)	91.5 (95% CI: 87.2–95.8)	98.7 (95% CI: 96.5–100)	98.0 (95% CI: 95.3–100)	94.4 (95% CI: 91.4–97.5)	95 (95% CI: 91–99)
Soft Voting Classifier	93.8 (95% CI: 91.5–96.1)	91.5 (95% CI: 87.2–95.8)	98.7 (95% CI: 96.5–100)	98.0 (95% CI: 95.3–100)	94.4 (95% CI: 91.4–97.5)	95 (95% CI: 91–99)
**SarcoNet** **(Proposed)**	93.8 (95% CI: 89.0–97.1)	91.5 (95% CI: 85.6–96.0)	98.7 (95% CI: 94.5–100.0)	96.9 (95% CI: 92.4–99.4)	92.1 (95% CI: 86.9–96.0)	0.94 (95% CI: 0.91–0.97)

**Table 6:** Ablation study of SarcoNet design.

Configuration	Accuracy (%)	Sensitivity (%)	Specificity (%)	Precision (%)	F1-Score (%)	AUC
4 layers, ReLU, Adam (0.001), Dropout 0.3	86	80	90	83	81.4	0.82
5 layers, ReLU, Adam (0.001), Dropout 0.3	89	83	92	87	84.9	0.86
6 layers, ReLU, Adam (0.001), Dropout 0.3	91	86	94	89	87.5	0.90
**6 layers, ELU, Adam (0.001), Dropout 0.3 (Proposed)**	**94**	**86**	**100**	**100**	**92.4**	**0.94**
6 layers, ELU, RMSProp (0.001), Dropout 0.3	90	85	92	88	86.5	0.88
6 layers, ELU, Adam (0.001), Dropout 0.2	91	85	95	90	87.3	0.91
6 layers, ELU, Adam (0.001), Dropout 0.5	88	81	91	85	83.1	0.87
6 layers, ELU, Adam (0.001), No BatchNorm	90	82	93	87	84.4	0.89

## Data Availability

The raw data supporting the conclusions of this article will be made available by the authors on request.

## Supplementary Materials

The following supporting information can be downloaded at: https://www.mdpi.com/article/10.3390/diagnostics15192513/s1, Table S1: TRIPOD+AI Checklist for SarcoNet Study.
