# Supplementary material for: SarcoNet: A Pilot Study on Integrating Clinical and Kinematic Features for Sarcopenia Classification
Source: Diagnostics (Basel). 2025 Oct 3;15(19):2513. doi: 10.3390/diagnostics15192513 (PMC12523922; doi:10.3390/diagnostics15192513)
Supplement: Supplementary file 1 [file diagnostics-15-02513-s001.zip › diagnostics-3830027-supplementary.pdf]

Table S1. TRIPOD+AI Checklist for SarcoNet Study

TRIPOD+AI Item	Description	How Addressed in This Study
Title and Abstract	State that the study develops and validates an AI model for diagnosis/prognosis.	Title and abstract explicitly mention SarcoNet, an ANN-based classifier for sarcopenia diagnosis.
Background and Objectives	Explain the medical context, rationale, and objectives.	Introduction highlights clinical burden of sarcopenia and the need for AI-based diagnosis. Objectives clearly stated.
Data Sources and Participants	Describe dataset origin, inclusion/exclusion criteria, and sample size.	Methods specify 30 participants, physician assessment, inclusion and exclusion criteria.
Outcome Definition	Define outcome to be predicted (diagnosis/prognosis).	Outcome defined as classification into sarcopenic vs. non-sarcopenic groups.
Predictors	List predictors used in model development.	31 clinical features (BCA, handgrip, TUG, 6MWT, chair stand) and 10 kinetic gait features reported in detail.
Missing Data	Explain handling of missing data.	Mean substitution used in this pilot; limitation acknowledged; advanced imputation planned for future work.
Model Development	Describe model type, architecture, and training process.	SarcoNet architecture (6-layer ANN with batch normalization and dropout) fully described. Classical ML baselines compared.
Model Validation	Describe validation methods and test-train split.	Stratified 70–30 split with 5-fold cross-validation performed.
Performance Measures	Report metrics with definitions.	Accuracy, sensitivity, specificity, precision, F1-score, and AUC all reported.
Model Explainability	Describe any interpretability methods applied.	Not performed due to small dataset; SHAP/LIME planned for future work.
Results	Present results clearly with tables/figures.	Results reported in Table 2 and supplementary ablation study.
Discussion of Limitations	Address dataset size, generalizability, and bias.	Discussion highlights small sample size, need for stronger baselines, and plans for expansion.
Future Work	Outline next steps and improvements.	Plans include larger datasets, advanced baselines (XGBoost, TabPFN), longitudinal data, and interpretable AI.
